# Investigating perspectives on e-health interventions to enhance maternal mental well-being: Results of a stakeholder interview

**DOI:** 10.1371/journal.pdig.0000326

**Published:** 2023-08-23

**Authors:** Juliane Schmidt-Hantke, Corinna Jacobi

**Affiliations:** Department of Psychology, Technical University Dresden, Dresden, Germany; Iran University of Medical Sciences, IRAN (ISLAMIC REPUBLIC OF)

## Abstract

Peripartum mental disorders are highly prevalent conditions and associated with adverse outcomes for the mother, the infant and the whole family. Despite the high burden, help-seeking is low. E-mental health interventions could overcome help-seeking barriers and thus improve perinatal mental health. However, usage and adherence are rather low. This study aims to assess attitudes and needs of different stakeholder groups and to identify relevant topics to develop an intervention meeting the needs of pregnant and postpartum women and thus, increasing utilization and adherence. Therefore, semi-structured interviews and focus groups with pregnant women (n = 3), mothers (n = 4), women who have suffered from a postpartum mood disorder in the past (n = 5), gynaecologists (n = 5), and midwives (n = 5) were conducted. All interviews were audio-recorded, transcribed verbatim, and analyzed using a thematic analysis. Almost half of the stakeholders reported previous experiences with e-mental health services. Anonymity, flexibility, promoting help-seeking, or bridging waiting-time for treatment as usual were regarded as the main benefits. Concerns regarding the usefulness of e-mental health interventions, absence of face-to-face contact or lack of integration into routine care were considered as barriers. With regard to the desired program content, six main topics were identified: pregnancy and puerperium, peripartum mood swings and disorders, support options, self-care, partnership, and fatherhood. Regarding preferred characteristics of e-mental health programs, stakeholders mentioned customizability, individual guidance, evaluation of user-feedback and continuous adoption, as well as a responsive and user-friendly design. Overall, online interventions for perinatal mental health were mainly considered as beneficial. Stakeholders underlined the high need for education regarding the use and effectiveness of e-mental health, to overcome concerns and obstacles and improve acceptability. Furthermore, developing customizable and individually-guided interventions were considered as promising to increase utilization of and adherence to e-mental health interventions.

## Author summary

Postpartum depression (PPD) is a common peripartum mood disorder and occurs in 10–15% of new mothers after giving birth. PPD can cause symptoms like depressed mood, fatigue, loss of interest, sleep disturbances, lack of concentration, anxiety, and suicidal thoughts, but can also have far-reaching consequences for the infant (such as internalising and developmental disturbances) and the whole family system. Due to a lack of social education on such peripartum mood disorders, the absence of a routine screening, as well as maternal feelings of shame and guilt, many affected mothers go undiagnosed and therefore untreated. The high prevalence rates and negative consequences of peripartum mood disorders emphasizes the urgent need for prevention. To overcome help-seeking barriers and to provide low-threshold and anonymous support, electronic mental health (EMH) services may be a feasible option. EMH services are internet-based and computerised services offering, for example, prevention, treatment, or aftercare. As peripartum-specific interventions are rare and the usage of existing interventions often remains low, a program fitting to the needs and circumstances of pregnant women and new mothers should be developed. Therefore, as a first step, stakeholder interviews will be conducted to assess perspectives and needs on peripartum-specific e-mental health interventions in Germany.

## Introduction

Pregnancy and postpartum period are considered to be times of well-being and happiness. However, transition to motherhood is also characterized by challenges through physiological and psychosocial changes. Approximately 50% to 70% of new mothers experience mood disturbances after having given birth, also known as “postpartum blues”, with a peak around the fourth day after delivery [1]. Besides these mild mood swings, a considerable number of women is affected by syndromes of postpartum depression. Postpartum depression is the most common postpartum mental disorder with a global pooled prevalence of 17.7% [2] and an increasing course over the first ten weeks after giving birth [3]. Based on recent research, 10% of women showed symptoms of depression already during early pregnancy [4]. Apart from peripartum depression, also peripartum anxiety disorders are common with a postpartum prevalence of 17.1% [5]. For antenatal depression and anxiety disorders, a prevalence of 9.3% has been reported [6].

Peripartum mood disorders are also associated with adverse birth outcomes, such as low birth weight or preterm birth [7], and can have far-reaching consequences for the mother-child interaction [8], child development [9], and the whole family system. Furthermore, 30% of women suffering from postpartum depression in community samples and 50% of affected women in clinical samples showed continued symptoms of major depression during and after the first year of life of their baby [10].

Despite the high burden of peripartum depression, the majority of affected women go undiagnosed and do not receive professional treatment [11]. This is partly due to a low mental health literacy of peripartum depression [12, 13]. Further barriers to treatment are time constraints, shame and perceived stigma [12, 14–16], fear of losing the baby [17], worries about medication in breastfeeding mothers [15, 16], as well as a lack of motivation [18]. Further obstacles are lacking access to support systems in rural areas [19] and–during the COVID-19 pandemic–a limited availability of face-to-face treatments.

It is therefore important to focus on prevention and early intervention, and to identify and tackle obstacles in help-seeking. E-mental health interventions can help to overcome many barriers as they provide low-threshold access, anonymity and flexibility, and a more cost-effective alternative to face-to-face treatment [20, 21]. In times of restrictions due to the pandemic and the associated limited access to face-to-face support, these interventions have proven to be particularly beneficial [22]. Previous research found mobile- and Internet-based health interventions to be effective in improving perinatal (pregnancy and the year following childbirth) mental health and reducing depression scores [23–26] with moderate (d = 0.55) to large (d = 1.03) effect sizes [27], which, however, differ between interventions providing human support (guided interventions) and unguided interventions (pooled effect sizes of d = 0.78 and d = 0.36, respectively) [28]. Promising results in reducing symptoms of depression were also reported in trials comparing e-health interventions to treatment as usual [17, 29, 30]. Thereby, existing research showed a significant reduction in outcomes like stress and anxiety as well [31], mainly in the postpartum period [29, 32]. Several studies have also demonstrated the effectiveness of preventive interventions for depression [33–35], especially in women at-risk [36–38]. As reported in former trials, participants perceived perinatal e-mental health interventions as useful and acceptable [23, 37, 39] with satisfaction rates between 86–95% [17].

Despite proven effectiveness and perceived advantages of web-based interventions, existing research shows high dropout rates [36, 40], with higher attrition in unguided compared to guided conditions [28, 41]. This is in line with findings of higher intervention adherence in guided [42] compared to unguided internet-based interventions targeting affective disorders in perinatal women [43]. A limiting factor for adherence may be the flexible, non-binding character of e-mental-health interventions compared to face-to-face interventions, especially in unguided conditions. Thus, flexibility can also be an obstacle as it can result in reduced interest and motivation. Perceived stigma, uncertainty about the treatment effectiveness and possible previous negative experiences are mentioned as additional barriers [43]. As further disadvantages, women stated the lack of face-to-face contact [44] as well as scepticism about opening up towards a stranger [45]. To increase acceptance and adherence, developing customizable interventions fitting women’s needs and individual circumstances seems to be promising [36, 40, 46]. In general, women stated to prefer digital maternal care services that are trustworthy and valuable [47], providing personalized, encouraging support and customizable, interactive content [41] on topics such as maternal well-being, information on pregnancy, child development or parenting [46].

In addition to the considerable attrition-rates observed in e-mental-health studies, also usage among healthcare providers is low, leading to a limited integration into routine practice [48]. This may be due to healthcare professionals´ scepticism toward e-mental health services, as these services were often only perceived as an additional support option, e.g., for specific patient subgroups [49]. Since the beginning of the COVID-19 pandemic, accompanied by an increased provision and usage of internet-based treatments as well as a decreased number of provided face-to-face treatments, perspectives of both professionals and patients have changed [50]. As recently reported, psychotherapists mentioned relatively positive attitudes towards web-based therapy. Concerns were raised about technical problems or recognizing patients’ emotions. When asked about the therapeutic relationship, many psychotherapists (65.3%) reported no changes compared to the pre-pandemic period [51]. Considering pandemic-related changes in patients’ experiences, mainly positive (62.8%) impressions were reported [51]. This is in line with previous, pre-pandemic research reporting high client-rated therapeutic alliance-scores in e-mental health interventions, that correspond roughly to those in face-to-face interventions [52]. Besides this, the development of a positive therapeutic relationship was also found in unguided e-mental health interventions [53]. Disseminating these findings and improving public knowledge about e-health services seems to be helpful in order to develop positive attitudes [54] and therefore to enhance acceptance and use of e-mental health interventions.

To the best of our knowledge, no previous study conducted a qualitative study evaluating the needs and attitudes of relevant stakeholders with regard to e-mental health interventions for the peripartum period in Germany yet. As mentioned above, studies conducted in other countries mainly failed to consider stakeholders’ views on disorder-specific e-mental health interventions. Additionally, before conducting this trial, no German e-mental health intervention for the peripartum period existed and thus, no data was reported on user satisfaction, acceptance and useful intervention components. We therefore assessed stakeholders´ experiences with and attitudes towards e-health interventions for improving peripartum mental health as a first step in developing a tailored Internet-based self-help intervention to enhance peripartum well-being and to prevent postpartum depression.

## Methods

### Study design

In order to develop an acceptable, user-friendly and effective e-mental health program for improving peripartum psychological well-being, a comprehensive overview on requirements from various angles is mandatory. Thus, experiences, attitudes and needs from different stakeholder groups were assessed using a qualitative, exploratory design. Therefore, focus group interviews, based on a semi-structured interview guide, and, if not feasible, semi-structured single-interviews were conducted. In agreement with the local ethics committee, an ethical approval was not required for this purpose.

### Participants

1. The first stakeholder group consisted of pregnant women and mothers, as they are potential users of e-mental health programs to support peripartum well-being and prevent postpartum depression and anxiety. 2. The second stakeholder group consisted of mothers who had suffered from postpartum depression or anxiety in the past. 3. As a third group, gynaecologists and midwives were interviewed as they are health care professionals and primary medical contact partners during pregnancy and postpartum. Therefore, they can provide extensive knowledge, are highly experienced with these conditions and act as potential facilitators of e-mental health programs for the peripartum period.

### Recruitment and procedure

Recruitment took place in June and July 2018. Pregnant women and mothers were recruited by distributing leaflets in nurseries, gynaecological and midwifery practices as well as social media postings. Health care professionals (gynaecologists and midwives) were recruited via e-mail and telephone. Eligible participants had to be over 18 years of age, German-speaking and belong to one of the above-mentioned stakeholder groups. As recommended [55], focus groups consisting of at least five participants were planned. The first focus group, consisting of midwives, was carried out by the first author and one undergraduate student (A.R.). Two further focus groups, one with primigravid women and one with mothers, as well as the single interviews with gynaecologists and women diagnosed with postpartum depression in the past were conducted face-to-face by A.R. The single interviews were conducted instead of the planned focus group interviews due to personal preferences of these two stakeholder groups. The interviews were video- or audio-recorded after receiving informed consent for interview participation and recording. Participating health care professionals received a reimbursement of 25 Euro after completing the focus group or interview. The remaining interviewees received a reimbursement of 20 Euro for their participation.

### Instruments

Semi-structured interview guides were developed for both the focus groups and the single interviews. Each interview started with a round of introductions, followed by 20 to 26 open-ended questions, depending on the respective stakeholder group. The questions addressed previous experiences with e-mental health programs, attitudes and expectations, desired program content and features, as well as potential obstacles about e-mental health interventions to support peripartum psychological well-being and to prevent postpartum depression and anxiety.

### Data analysis

**Fig 1:**
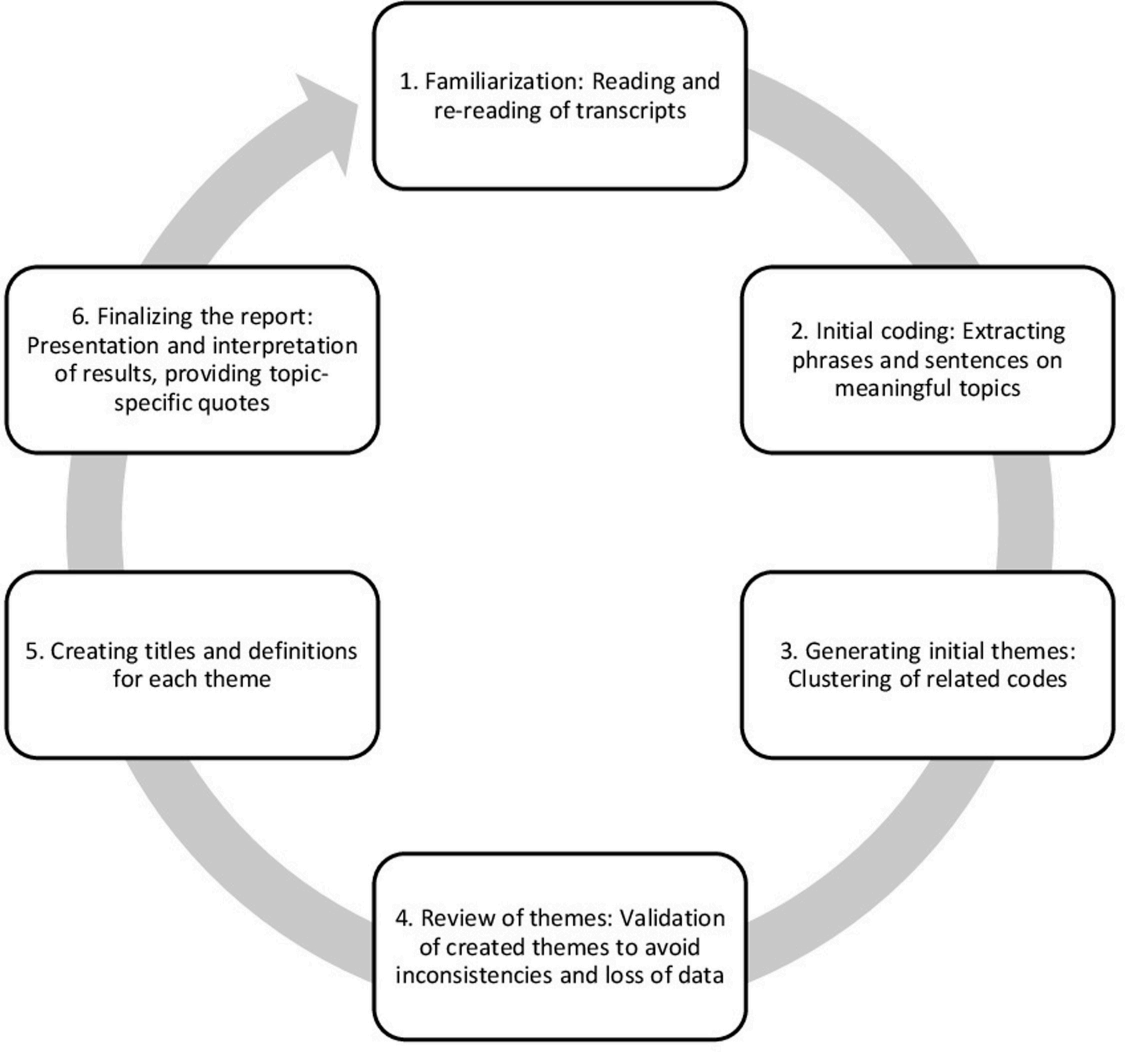
Steps of the thematic analysis (adapted from [56]).

After conducting the interviews, the video and audio files were transcribed by an undergraduate student. In the subsequent step, a thematic analysis following Braun and Clarke was conducted (Fig 1) [56]. This six-steps method enables the identification, analysis, and documentation of themes of relevance for the research question. First, all transcripts were read by two independent coders (J.S.H. and A.R.) to become familiar with the data and to create initial ideas on core topics. In a second step, initial codes for interesting data were generated. Each code indicates an interesting feature at a semantic level. In a third step, codes of data with meaningful coherence were assigned to overarching themes linked to the topics of the interview guide. These themes were critically reviewed in step four. If necessary, re-coding took place. Fifthly, all themes were clarified and definitions for each theme were specified. Furthermore, useful sub-themes were defined. The sixth step included the final analysis, choosing appropriate examples and writing the report.

Results of the thematic analysis will be presented based on the structure of the interview-guide as well as on subcategories emerged in the course of the thematic analysis. Due to the large accordance in statements given by the different stakeholder groups, results will be presented across groups. To illustrate the results, selected quotes will be embedded.

## Results

Stakeholders’ experiences, attitudes and needs were assessed by conducting three focus groups and ten single interviews (Table 1). The first focus group was conducted with female midwives (MW), the second with primigravid women (P) and the third one with mothers (M). Additionally, 3 male and 2 female gynaecologists (G) as well as 5 women who have suffered from postpartum depression in the past (W) participated in single interviews. The total interview duration was 07:40:59 (hh:mm:ss). Due to short-term cancellations, the intended sample size of at least 5 participants was not reached in focus groups with pregnant women and mothers.

**Table 1:** Overview on stakeholder groups and interview characteristics.

Stakeholder group	Number of interviewees (N)	Interview method	Interview duration (mm:ss)
Primigravid women (P)	3	Focus group	50:24
Mothers (M) Number of children 1 2	4 3 1	Focus group	55:16
Women diagnosed with postpartum depression in the past (W) Number of children 2	5 5	Semi-structured single interviews	29:29–44:34 (mean: 34:37, SD: 05:49)
Midwives (MV)	5	Focus group	47:57
Gynaecologists (G)	5	Semi-structured single interviews	24:05–32:38 (mean: 26:52, SD: 03:22)

The following results are presented along the research questions, identified main themes and subcategories, as well as across groups, as there was a notable content-related accordance between the different stakeholder groups. Table 2 gives an overview of the different categories, identified themes and reported aspects.

**Table 2:** Overview on the categories, identified themes and reported aspects resulting from the thematic analysis (based on [56]).

Category	Definition	Reported aspects
Advantages	Perceived benefits of e-health services	‐ easy, anonymous, and low-threshold access ‐ temporal flexibility ‐ geographical flexibility ‐ immediate availability ‐ bridging waiting-time ‐ reducing professionals’ workload ‐ promoting help-seeking behaviours
Disadvantages	Perceived disadvantages of e-health services	‐ lack of personal contact and therefore possible communication barriers ‐ considered to be appropriate only up to a limited symptom severity
Topics	Preferred topics of e-mental health interventions to promote peripartum mental health	‐ basic pregnancy and puerperium related issues, e.g., mother-child-interaction ‐ peripartum mood fluctuations and mental disorders ‐ support options ‐ self-care ‐ partnership ‐ fatherhood
Characteristics	Preferred characteristics of e-mental health services	‐ preferred device: app (N = 3), website (N = 2), both (N = 11), no preference (N = 6) ‐ customizability ‐ accounting for user feedback ‐ guided self-help ‐ contact option ‐ usability ‐ reliable and useful content
Obstacles	Perceived barriers to the use of e-mental health interventions	‐ acceptance of e-mental health interventions ‐ unwillingness to access e-mental health services ‐ concerns regarding the effectiveness of e-health interventions ‐ no perceived need for treatment due to a lacking awareness of postpartum mental disorders ‐ inability to use e-mental health interventions due to high symptom severity ‐ technical obstacles ‐ lack of time ‐ dissemination and integration of e-mental health interventions in routine care

### Experiences with and attitudes on e-mental health interventions

#### Experiences

When asked about their experiences with e-health services, 12 out of 22 participating stakeholders mentioned to have no previous experiences. One pregnant woman stated to use a meditation app and 5 out of a total of 9 interviewed mothers reported experiences with pregnancy- and motherhood-related apps, websites and forums. One midwife reported experiences with an online-tool addressing ADHD, one gynaecologist noted previous experiences with an online-course, and one gynaecologist mentioned to support clients also via e-mail.

#### Perceived advantages

A factor commonly perceived as beneficial is the *easy*, *anonymous*, and *low-threshold access* to e-health services. Additionally, participants stated *temporal flexibility* as an advantage of particular importance during pregnancy and early motherhood, as women have to deal with many new responsibilities. According to this, being *geographically flexible* was also mentioned as a main benefit. Women may use the program wherever possible, even in underserved, rural areas. As a further advantage, the *immediate availability* of e-health services was underlined. Therefore, e-health programs were perceived as an opportunity to *bridge waiting-time* for face-to-face treatment.

*“It is definitely an advantage that it is simple and easily available and offers low-threshold access*. *… Also in underserved … structurally weak regions*.*” (G5)*

*“It is an advantage*, *if you are living outside like me and can´t quickly move somewhere*.*” (W3)*

*“An advantage is to receive immediate support*, *even when you don´t dare to leave the house*.*” (W4)*

*“For many people*, *threshold seems to be low*.*” (P2)*

*“It provides anonymity*. *You don´t have to come out*.*” (G5)*

*“The advantage is that it is anonymous*. *… You are ashamed*.*” (W1)*

Some gynaecologists and midwives added the benefit of e-health programs to *reduce professionals’ workload* by overtaking tasks like psychoeducation, early detection of risk-status or providing early information on support options. Some stakeholders of both groups also pointed out that e-health services can *promote help-seeking behaviours*, e.g., by providing recommendations for action.

*“I could imagine to say ´Look at this program and work through it*. *Afterwards you can come back to me*. *`… It is rather supportive*.*” (G4)*

*“Actually*, *we should have the claim to cover the function of the App ourselves*. *In this respect*, *it is a relief*.*” (G1)*

The typical non-obligatory character of most e-health interventions was considered as being both beneficial and adverse.

*“On the other hand*, *it is of course also difficult because there is no motivation*. *If I have an appointment with someone*, *… motivation is greater as if I do something online on my own*.*” (MW1)*

#### Perceived disadvantages

Due to the *lack of personal contact*, all interviewed stakeholder groups considered e-health services to be only of *limited usefulness*, e.g., to provide additional support or bridge waiting time. Participants emphasized that these interventions are *unable to replace face-to-face support* for various reasons: First, stakeholders expected the lack of in-person conversation to affect interactions between users and professionals due to an impaired opportunity to respond to both users’ verbal statements and non-verbal reactions. Second, professionals and mothers considered e-health interventions to be only applicable to a certain degree of symptom severity. One gynaecologist stated difficulties in terms of preventing social isolation in women being already affected from a peripartum mental disorder. According to this, a mother stated “Even when you´re using the app, you´re still alone.”

Overall, participating stakeholders noted more advantages than disadvantages.

*“It can´t keep you from isolation*.*” (G5)*

*“It is not suitable for severe cases*.*” (G5)*

*“The program is just supportive*. *But I think*, *a personal conversation is something completely different from self-treating through clicking on something*. *… It certainly helps*, *but I think only to a certain degree*.*” (P2)*

*“I think that an app is unable to compensate for a personal story*. *It is something different to face a human instead of an app*.*” (M3)*

*“It is a source of information*, *but it is not a personal contact*.*” (MW2)*

*“When you feel really bad*, *I think you have to go somewhere where you have a person in front of you*.*” (W3)*

### Preferred topics of e-mental health interventions to promote psychological well-being during pregnancy and postpartum

All participants were asked to point out required and desired topics of an e-health intervention to promote peripartum psychological well-being. This question also raised discussions on the most appropriate time around pregnancy to provide the program. Midwives perceived early pregnancy as the most useful period, whereas some potential users (pregnant women, mothers, and women with a postpartum depression in the past) and gynaecologists considered a later stage of pregnancy to be the best period, e.g., based on assuming an increasing interest in this program over the course of pregnancy. One midwife suggested to distinguish between pregnancy and puerperium and to provide two different program versions.

Overall, 6 main topics were identified. The topics include 1. basic pregnancy and puerperium related issues, 2. peripartum mood fluctuations and mental disorders, 3. support options, 4. self-care, 5. partnership, and 6. fatherhood. All themes are described below.

First, general **pregnancy and puerperium related themes** such as mother-child-interaction and bonding or stages of child development as well as bureaucratic issues were perceived as essential. This topic should also focus on possible upcoming challenges like developing a new daily routine (M) or breastfeeding problems (W), and how to deal with them.

*“It has to support me in terms of questions about pregnancy and birth*.*” (W2)*

*“I can imagine that you click on that and then you can specify your needs and see that there are … tools on how to deal with the baby*.*”(G5)*

Second, gynaecologists, primigravid women as well as women being diagnosed with postpartum mood disorder in the past mentioned the need to address **peripartum mood fluctuations and mental disorders**. They suggested to highlight the high prevalence of mood changes including also negative thoughts and feelings during pregnancy and postpartum. One gynaecologist said: “Being a mother isn´t always rosy.” In this context, mothers previously affected by a postpartum mental disorder emphasized the desire for authentic case examples that bolster mothers with messages like “Don´t be ashamed.”, “You´re not alone.”, or “It will get better, there is a way out.” Stakeholders (G, W) underlined the importance of taking care of maternal feelings of guilt and shame because these feelings often lead to a reduced help-seeking behaviour. All stakeholders stated the need for a “warning signs-” and symptom checklist to recognize changes and facilitate early help-seeking. Furthermore, professionals and pregnant women suggested to avoid labelling the program as an intervention concerning postpartum mental disorders. Since many women are afraid of being diagnosed with a postpartum mental disorder and therefore avoid confrontation with this topic, a label may act as a deterrent and lead to reluctance.

*“First of all*, *it has to provide education during pregnancy*. *… That there are opportunities to talk about it …*. *And to inform about the first symptoms during the postpartum period and that you can get into something unpleasant*. *That a woman … does not think ´I´m wrong*, *there is something wrong with me*, *I´m a bad mother*.*`… That this can be a first symptom*.*” (MW5)*

*“There should definitely be information on symptoms*. *… In any case I would include pictures of women affected and cite them*: *I also had this*. *But it is gone*. *I have sought help*.*” (W1)*

The third issue was related to **support options** during the peripartum period. The pregnant women, professionals and mothers affected by a postpartum mental health disorder in the past considered lists of on-site assistance and local self-help groups as essential.

*“To provide information on local contact points I can turn to*.*” (P1)*

*“It is essential to include contact points*.*” (W1)*

**Self-care** was noted as a fourth main topic by all stakeholder groups. The program can help to identify and satisfy own needs (G). Potential ideas on how to promote self-care ranged from relaxation and breathing techniques to mindfulness exercises and advices on nutrition and sleep.

*“… because then you could really say this is a wonderful app that allows you to understand your needs*, *to see what possibilities there are*.*” (G1)*

*“… there are relaxation techniques*, *… autogenous training*, *how can I find sleep*.*” (G5)*

Fifthly, gynaecologists considered **partnership** as another important issue. This topic should deal with changes in partnership, effective communication and how to involve the partner in new responsibilities.

“How can I get my partner involved? What kind of network can I build?” (G5)

Additionally, all stakeholder groups suggested to provide an optional **module for fathers**, e.g., addressing recommendations how to support their partner during the peripartum period, but also father´s psychological well-being and self-care. At this point, gynaecologists noted that also fathers can develop a postpartum mental disorder.

*“Also for the partners*. *They are falling through the cracks*. *And there is also postpartum depression in men*.*” (G5)*

### Preferred characteristics of e-mental health services

#### Mobile app versus website

Three (P, W, G) out of 22 interviewed stakeholders stated to prefer the program content being delivered via an app, especially for younger women. Two participants (MW) would prefer a website, 11 (P, W, G, MW, M) participants were in favour of both options, and 6 did not provide answers. One reason mentioned for preferring an app-version was the increased flexibility. For instance, one mother with a postpartum diagnosis in the past said “How often do you have the possibility to use a computer?”.

*“I think*, *an app is probably useful*. *… It’s just that the phone is always there*. *And*, *yes*, *I find it more practical on the cell phone than opening a website*.*” (P3)*

*“I would probably rather use a website*. *But I think*, *it is age-related*.*” (W2)*

*“And that it is not just accessible via the computer but also as an app*.*” (W5)*

#### Customizability

All stakeholder groups emphasized the need for a customizable content based on user needs. There was also a desire for a modular design consisting of short sessions with a maximum duration of ten minutes each (MW). In addition, pregnant women and mothers preferred a combination of predetermined as well as optional sessions. Users should be able to follow these modules according to their individual time-plan and preferences.

“I think rather individually, that you can see: Ok, what do I really need right now? Because when being stepwise, there is a risk of having steps in between that you kind of don’t like so much or something, and that the next step only gets unlocked when you’ve completed this one.” (P3)

*“I would say that the woman can decide for herself depending on her available time …*. *And at most ten minutes*, *so that it can be accessed when the child is relaxed*.*” (MW3)*

#### Assessment of user feedback

A further issue to be considered is the evaluation of user feedback. One gynaecologist recommended to assess user feedback in order to allow required program adaptations.

*“You can’t develop something like that*, *put it down and that´s it*. *It is a process*. *The families are given the opportunity to provide feedback*. *… That´s a dynamic” (G2)*

#### Combination of self-help and external feedback

When asked about how to provide the topics mentioned above, stakeholders suggested to combine interactive self-help elements and external support. The latter refers to individual feedback provided by a professional, and a group chat function. Three out of five midwives and one pregnant woman argued against a group chat, the remaining stakeholders would have appreciated a group chat that is monitored and moderated by a professional, to avoid, e.g., misinformation or harmful advice. Self-help components should contain psychoeducation and exercises, e.g., on relaxation or mindfulness.

*“There is a level of information and a level of care … so you could offer self-help*, *external help and group-help*. *In the group-help*, *there could be moderated forums where people can exchange information*. *In the external help*, *there could be a contact up to an individual chat system with a professional psychotherapist*. *And the self-help could include simple exercises*.*” (G1)*

#### Contact option

Providing users with the option to get in touch with a professional via e-mail or phone was perceived as a must-have by all participants. Gynaecologists added the suggestion to include a short description and a picture of the contact person to promote confidence-building. The focus group consisting of mothers underlined to ensure anonymity when offering a contact option.

“There could be a contact up to an individual chat system with a professional psychotherapist.” (G1)

#### Usability

In general, all stakeholder groups emphasized the need for an appealing and trustworthy design. In addition, there was a demand for usability. To meet this demand, midwives and mothers stated to include a simple structure, clear and comprehensible user instructions, and special features like a keyword search as well as the possibility to display previously logged data.

*“… that appeals to you in terms of design*. *That … the design fits into today´s world*.*” (M2)*

#### Perceived usefulness

Stakeholders underlined the importance of providing reliable and useful content delivered by different modalities to promote user engagement. To avoid information overload in text passages, midwives suggested to include read-more elements as well as links to further topics. Additionally, there was a desire for audios and explanatory videos, taking account of an ensured accessibility, e.g., through embedding subtitles. A factor considered as fundamental by professionals was to use a positive and encouraging wording and to avoid fear-inducing or deterrent language.

*“… videos are also conceivable*, *explanatory videos*.*” (M1)*

*“That you provide it in form of a video*, *but still include a summary below*. *If you say*: *Okay*, *I prefer to read through it now*, *because I don’t want any votes now*, *I don’t want to see anything now*, *I just want to have my peace and quiet*.*” (M2)*

### Obstacles to be considered

When asked about possible obstacles, participating stakeholders examined some external and internal factors.

First, *acceptance of e-health* programs and *willingness to use* them were addressed as potential internal barriers. Both focus groups consisting of midwives and pregnant women pointed out to avoid introducing e-health programs as a treatment, but to offer it as an option to provide additional support. Two mothers who had suffered from a postpartum mental disorder stated a *lack of trust regarding the effectiveness* of e-health interventions. According to this, a low *perceived usefulness* was mentioned by three gynaecologists. A fourth gynaecologist stated e-health interventions to be only effective in combination with face-to-face support as part of blended care.

*“So*, *I wouldn’t trust the program to the point that I would totally put that on a level*. *By facilitating access or as a supportive help alongside counselling*, *if really necessary*, *I would fully accept it*.*” (P1)*

Besides this, women previously affected by a postpartum mental disorder mentioned a *lacking awareness of postpartum mental disorders* as a further hindering factor. If not recognizing the *necessity for support*, an affected woman would not use the proposed e-mental health program. In addition, a high *symptom severity* was considered to impede help-seeking.

*“Or maybe address this topic at the childbirth preparation course*. *Postpartum depression ‐ and if you´re affected*, *please contact …*. *And of course*, *not everyone will jump on it*, *because you really assume that it doesn’t affect you*. *That’s what I thought*, *too*. *But that at least you have heard that it affects so many people*.*” (W1)*

With regards to external barriers, *technical obstacles* like a poor Internet connection in rural areas or a *lack of time* due to responsibilities during the postpartum period were mentioned by the focus group consisting of mothers.

Another issue raised was the *dissemination and integration* of e-health interventions *in routine care*. In general, stakeholders emphasized the need for promoting the program, preferably by gynaecologists and midwives or in prenatal classes. Additionally, they suggested to deposit information leaflets inside the maternity records.

## Discussion

### Principal findings

This study served as the first step toward the development of a web-based intervention to prevent postpartum depression and anxiety, aimed to be delivered to pregnant women in the third trimester. To meet maternal needs and preferences, this study examined the perspectives of five different stakeholder groups: pregnant women, mothers, women with a history of a postpartum mood disorder, midwives and gynaecologists.

More than half of the interviewed stakeholders reported previous experiences with web-based mental health services. Similarly, more than half of the mothers reported the use of perinatal care apps. This is in line with previous studies stating a high usage of pregnancy- and childbed-related websites and apps in peripartum women [17]. One out of five interviewed gynaecologists reported to provide additional client-support via e-mail. This low proportion reflects the often reported hesitant usage of e-mental health tools in routine care [57] before the COVID-19 pandemic.

In general, all stakeholders noted more anticipated advantages than disadvantages in e-health interventions. They highlighted many of the well-documented benefits, such as low-threshold access, flexibility and anonymity. E-health interventions are therefore not only beneficial to overcome help-seeking barriers like shame and fear of stigma, but also to meet time restrictions experienced by the majority of new mothers [12, 14–16]. As a further benefit, the immediate availability was mentioned. This is beneficial in terms of offering support in rural or underserved areas. In addition, it was perceived as an opportunity to bridge waiting-time for face-to-face treatment.

With regards to disadvantages, the majority of interviewees considered e-mental health to be merely complementary to face-to-face support. The most frequently mentioned drawback was the anticipated lacking personal contact in e-health interventions. This issue can be solved via offering human support, i.e., guidance, as an additional feature. Referring to this, concerns were raised, e.g., regarding the therapeutic relationship. These concerns could be countered by focusing more on education and training about e-mental health and, for example, how to build and promote the digital therapeutic alliance. As recent research suggested, many health professionals who provided web-based therapy during the COVID-19 pandemic experienced the therapeutic alliance as comparably strong as in face-to-face sessions [51]. With regard to user experiences of the therapeutic alliance in e-mental health interventions, previous studies reported high and nearly similar scores compared to those in face-to-face therapies [52]. A stable, emotional therapeutic alliance may also serve as a factor to overcome users concerns of being on their own when using an e-health intervention. Guidance was also found to be a factor promoting user engagement and reducing attrition [28, 41].

Besides the need for guidance via individual support by a professional, stakeholders perceived a moderated group chat as desirable, since it allows an exchange of experiences between (expectant) mothers. This could also improve user motivation.

A point raised by all interviewees is the demand for interventions being customizable and thus perceived as tailored and personalized. The intervention content should therefore fit users’ individual needs and circumstances, which is in line with previous research [36, 40, 46]. In addition, a desire for modular interventions consisting of short sessions was believed to enhance user engagement. According to this, a negative relationship between the number of program sessions and adherence was found in former studies [30]. A further factor that was mentioned to improve acceptance and satisfaction is a user-friendly, appealing intervention design. This includes e.g., a trustworthy design, clear structure, simple instructions, interactive components as well as multimedia components like pictures, audios or videos. The latter is consistent with findings from a previous study on an online-based intervention for postpartum depression, which report users´ request for multimediality [46].

In addition to the factors considered to improve user satisfaction, stakeholders stated their preferences for mode of delivery. Most stakeholders preferred to have both an app-based format and a browser-based format. A quarter of stakeholders preferred access through a website, the remaining stakeholders favoured a smartphone app. The latter offers an increased flexibility, since new mothers have few opportunities to sit in front of a computer. Previous studies reported that intervention content has mainly be accessed through the app when offering both formats [58]. Delivery via app is therefore considered to enhance user satisfaction and adherence [17, 40, 46], which should also be kept in mind when developing an e-mental health intervention.

Beside stakeholders’ preferences on the intervention characteristics, they were also asked about their preferences regarding the intervention content. In this context, the time of delivering the intervention has been discussed. Overall, stakeholders emphasized the importance of providing prevention already during pregnancy. Whereas midwives considered early pregnancy as the most useful period, the remaining stakeholders would prefer an advanced state of pregnancy. As figured out in previous studies, an antenatal onset of peripartum mental disorders has been reported [4], with an increased prevalence over the course of pregnancy [3]. This supports the statement of midwives to provide support as early as possible. In this case, pregnancy-related issues have to be addressed in the prevention program as well. In general, stakeholders emphasized the importance of six main topics to be included: 1. basic themes related to pregnancy and puerperium (e.g., mother-child-interaction), 2. challenges of pregnancy, peripartum mood fluctuations and mental disorders, 3. pre- and postpartum support options, 4. self-care, 5. partnership, and 6. fatherhood. Referring to the second issue, a prevention program is also supposed to signalize normality of unpleasant feelings or mood disturbances during this period, that affected women are not alone and, above all, to encourage support-seeking. Destigmatization may reduce feelings of guilt and shame [12] and therefore promote help-seeking behaviour. Overall, the abovementioned issues are related to a variety of risk factors being relevant for the development of peripartum mental disorders, such as lacking social support or marital dissatisfaction [59]. Addressing these issues already in pregnancy may decrease the risk to develop a peripartum mental disorder. Furthermore, providing information on relevant peripartum-related topics meeting women’s needs and circumstances seems to be promising in enhancing intervention acceptance and adherence [36, 40, 46] and thus, may contribute to improving maternal well-being.

Besides informing about peripartum mental disorders and the importance of prevention, it is necessary to provide information on available support options such as e-mental health, particularly in times of a pandemic or other situations with limited access to face-to-face health care. E-mental health interventions offer general advantages such as low-threshold access, anonymity, flexible use, or support in rural areas [20]. To increase uptake and use, stakeholders´ concerns (e.g., lack of trust in the effectiveness of e-health interventions or concerns regarding developing a therapeutic alliance) have to be addressed. Informing potential health care providers about the value, benefits and effectiveness of e-health services can help to overcome perceived barriers and to develop positive attitudes towards these interventions [54, 57]. As the present stakeholder interviews took place before the COVID-19 pandemic, it can be assumed that experiences with and attitudes on e-health interventions have changed during the past months. Against concerns about the therapeutic relationship, even pre-pandemic research has shown the development of a positive therapeutic alliance in e-mental health interventions [52, 53]. A recent study has shown that almost two thirds of psychotherapists have not perceived a deterioration compared to the alliance in pre-pandemic in-person sessions [51]. Additionally, it seems to be useful to inform health care providers about the patients’ point of view. During the COVID-19 pandemic, most patients experienced Internet-based psychotherapy as positive (62.8%) or neutral (27.6%) [51]. These findings as well as new experiences gained during the last few months may have led to an altered perspective on technology-based interventions in stakeholders interviewed in the present study.

### Limitations and future directions

In general, stakeholders´ attitudes usually depends on factors like personal e-health experiences, therapists clinical experience, or therapy modality [51]. As the latter factors were not taken into account in the present study, future studies may benefit from considering these determinants. Additionally, demographic variables like age, level of education of potential users, or residential environment (urban or rural) should be taken into account as these factors may also influence attitudes. For example, it is assumed that willingness to make use of e-health services is higher in underserved areas due to the poor supply of mental health care providers.

While interviewed stakeholders in our study had heard about e-mental health interventions before, more than half of them reported no personal experiences. Only one out of five pregnant women as well as five out of nine mothers reported a previous use of technology-based mental health services. Among health care professionals, only one stated to offer Internet-based support. Therefore, the answers to interview questions were mainly based on theoretical knowledge or assumptions. Against the background of previous research, it is expected that an increased experience will lead to more positive attitudes [49]. Furthermore, it should be noted that the present trial was conducted before the occurrence of the COVID-19 pandemic and may therefore not reflect the current, possibly changed perspectives of the abovementioned stakeholders. As many healthcare professionals and patients were forced to use e-mental health services during the past months [51], the percentage of users has increased considerably. The changed conditions do not only lead to altered attitudes and perspectives, but may also be accompanied by altered needs in peripartum women and health care providers. Thus, future studies have to assess stakeholders’ perspectives as well as changes in women’s needs and expectations during the pre- and postpartum period against the background of an increased availability and use of e-health services.

It should also be mentioned that one stakeholder group was not taken into account in the present study: psychotherapists providing support for women suffering from a peripartum mental disorder. As implementation of technology-based psychotherapeutic support seems to be easier compared to support provided by midwives and gynaecologists, it is expected that psychotherapists may have a different perspective and would highlight further advantages, disadvantages, and other valuable aspects. Therefore, this stakeholder group should be considered in future research.

## Conclusion

The results of this study suggest the benefits of e-health interventions, especially in times of a pandemic or other crisis, along with difficulties in providing usual face-to-face support. Besides this, technology-based support can help to overcome many peripartum-related barriers. Since affected women often experience feelings of shame and guilt, the anonymity of an e-health intervention can facilitate help-seeking behaviours. Furthermore, flexibility was perceived as an advantage as technology-based services can be accessed anywhere and at an individually favourable time. As new mothers face the challenge of developing a new daily routine fitting children´s needs, any further responsibility accompanied by fixed appointments can cause additional stress. Besides the mentioned benefits, the study also identified factors that stakeholders perceive as barriers to the use of e-mental health, e.g. the lack of confidence in their effectiveness. In this respect, it is important to consider these concerns in future research.

Overall, results of the present study emphasize stakeholders´ generally positive attitudes towards e-mental health services and support the development of customizable, tailored Internet-based interventions aiming to improve maternal mental well-being while meeting the specific needs of peripartum women.
